# Clinical and inflammatory features based machine learning model for fatal risk prediction of hospitalized COVID-19 patients: results from a retrospective cohort study

**DOI:** 10.1080/07853890.2020.1868564

**Published:** 2021-01-07

**Authors:** Xin Guan, Bo Zhang, Ming Fu, Mengying Li, Xu Yuan, Yaowu Zhu, Jing Peng, Huan Guo, Yanjun Lu

**Affiliations:** a Department of Laboratory Medicine, Tongji Hospital, Tongji Medical College, Huazhong University of Science and Technology, Wuhan, China; b Department of Occupational and Environmental Health, State Key Laboratory of Environmental Health (Incubating), School of Public Health, Tongji Medical College, Huazhong University of Science and Technology, Wuhan, China

**Keywords:** COVID-19, machine learning, fatal risk, extreme gradient boosting

## Abstract

**Objectives:**

To appraise effective predictors for COVID-19 mortality in a retrospective cohort study.

**Methods:**

A total of 1270 COVID-19 patients, including 984 admitted in Sino French New City Branch (training and internal validation sets randomly split at 7:3 ratio) and 286 admitted in Optical Valley Branch (external validation set) of Wuhan Tongji hospital, were included in this study. Forty-eight clinical and laboratory features were screened with LASSO method. Further multi-tree extreme gradient boosting (XGBoost) machine learning-based model was used to rank importance of features selected from LASSO and subsequently constructed death risk prediction model with simple-tree XGBoost model. Performances of models were evaluated by AUC, prediction accuracy, precision, and F1 scores.

**Results:**

Six features, including disease severity, age, levels of high-sensitivity C-reactive protein (hs-CRP), lactate dehydrogenase (LDH), ferritin, and interleukin-10 (IL-10), were selected as predictors for COVID-19 mortality. Simple-tree XGBoost model conducted by these features can predict death risk accurately with >90% precision and >85% sensitivity, as well as F1 scores >0.90 in training and validation sets.

**Conclusion:**

We proposed the disease severity, age, serum levels of hs-CRP, LDH, ferritin, and IL-10 as significant predictors for death risk of COVID-19, which may help to identify the high-risk COVID-19 cases.

**KEY MESSAGES**
1. A machine learning method is used to build death risk model for COVID-19 patients.
2. Disease severity, age, hs-CRP, LDH, ferritin, and IL-10 are death risk factors.
3. These findings may help to identify the high-risk COVID-19 cases.

## Introduction

The continuous pandemic of coronavirus disease 2019 (COVID-19) caused by severe acute respiratory coronavirus 2 (SARS-CoV-2) is showing an unprecedented attack to the global health system. Till 15 November 2020, the outbreak of COVID-19 had caused more than 53 million individuals infected and more than 1.3 million people dead globally; specifically, the U.S. and European countries, the most affected regions with increasing mortality, jointly account for over 47% of cases and 44% of deaths in the world [1]. The largest present report conducted by the Chinese Centre for Disease Control and Prevention with 72,314 cases revealed the average case-fatality rate was 2.3% [2], while a retrospective study among 379 critically ill adult patients have observed a striking day-28 mortality of 27%, ascending sharply with older age and for patients with latent comorbidities [3]. The death risk induced by severity of COVID-19 posed great pressure on medical service, resulting in a shortage of critical care resources and heavy disease burden.

To optimize the treatment and recovery of patients with the limited medical resources, it is of great significance to identify early detection prognostic biomarkers to discriminate COVID-19 patients who would probably develop critical illness and to evaluate their relevant mortality risk during the global pandemic. Prior study implemented in a development cohort of 1590 patients and a validation cohort of 710 patients has established a traditional risk score model by using 10 independent predictors including age, onset symptoms, comorbidities, and several laboratory findings [4]. During the recent global exigency, various machine learning (ML) and artificial intelligence (AI) technologies have been widely applied in patients tracing, vaccine development, and patients screening for its better scale-up, speed-up processing power [5]. However, few studies have focussed on the ML and AI utilization in discerning patient’s disease progress and estimating death risk.

Under these circumstances, we conducted a retrospective cohort study among 1270 COVID-19 patients admitted in Wuhan, China, aiming to use a mathematical model method based on interpretable ML algorithms to help discriminate significant death risk factors for COVID-19.

## Methods

### Study design

After excluding pregnant women and subjects with missing information about comorbidities, the 1270 COVID-19 patients admitted in two hospitals in Wuhan between 27 January and 5 April, 2020 were enrolled in our study. These patients were confirmed as COVID-19 according to diagnostic criteria established by WHO interim guidance by positive RT-PCR detection of nasal or throat-swab specimens. Among these patients, 984 were admitted in the Sino French New City Branch of Tongji Hospital in Wuhan with recruitment period ranging from 27 January to 5 April, 2020 and the 286 cases were admitted in the Optical Valley Branch of Tongji Hospital in Wuhan between 3 February 2020 and 26 March 2020. The illness severity of COVID-19 patients was classified into mild, moderate, severe, and critical according to the Diagnosis and Treatment of COVID-19 guidelines published by the Nation Health Commission of China [6]. In this study, we defined the mild and moderate type as non-severe group, while the severe and critical type were categorized as severe group.

### Data sources and processing

Electronic medical records of all patients were reviewed to collect the demographic information, clinical characteristics (onset symptoms, disease severity, and comorbidities), laboratory examinations (blood routine examination, cytokines and infection-related factors, blood coagulation factors, and serum biochemical index), and chest CT scan findings on admission. For some laboratory markers were below the limits of detection (LOD) among >15% subjects, we categorized these biomarkers as binary variables by using the normal reference range as cut-off value in the subsequent analysis. The dominating outcomes were discharge or decease. Computerised database was used to neaten the collected original data and further cross-check. Ethics approval for collection and analysis of all data from these patients was approved by the Ethics Committee of Tongji Hospital of Tongji Medical College, Huazhong University of Science and Technology (TJ-IRB20200201).

### Statistical analysis

Descriptive data were expressed as frequencies (%) for categorical variables and as medians with interquartile ranges (IQR) for continuous variables. Mann–Whitney *U* and Chi-square tests were conducted to estimate the differences of continuous and categorical data between surviving and not surviving groups, respectively. To optimize the latent collinearity and avert over-fitting of variables, the least absolute shrinkage and selection operator (LASSO) regression analysis was carried out in the training set of 984 patients to select the most significant clinical characteristics for mortality risk of COVID-19 by using the R software package “glmnet”. As described herein, 48 clinical features with missing values <20% were enrolled into the variable shrinkage process. The LASSO regression was established by using a Cox proportional hazard model, whose optimal value of λ with the minimum partial-likelihood deviance was selected by using 10-fold cross-validation [7].

Subsequently, variables selected by LASSO regression were entered into a high-performance ML prediction model namely XGBoost. The importance of candidate features in XGBoost is identified by its cumulated use in each decision step in trees [8]. To avoid model over fitting, we split the 984 cases randomly to training and internal validation sets in the ratio 7:3 (were recorded as training and internal validation sets, respectively) and then grid search method based on “caret” package in R software was conducted in the training set to tune XGBoost hyperparameters including number of trees (nrounds), the learning rate (eta), minimum loss to expend on a leaf node (gamma), maximum tree depths (max_depth), minimum sum of instance weight needed in a child node (min_child_weight), and subsampling proportion (subsample) [9]. The optimal hyperparameters was selected according to the minimum root mean square error (RMSE) in grid search process by using 10 repeats 10-fold cross-validation. The XGBoost model was finally trained in the training set with the following hyperparameter settings: max_depth = 3, eta = 0.1, gamma = 0.2, min_child_weight = 4, subsample = 1, and nrounds = 145, while all other hyperparameters were used as their default values. We defined this model as “multi-tree XGBoost” and the ranks of feature importance were then obtained.

To further select the most significant features related to mortality risk, 100-round 5-fold cross-validation was conducted in training and internal validation sets. The key features were identified according to the performances of the model with improved area under curve (AUC) score <0.5% when adding the seventh feature to the model. Finally, six features were selected as significant predictors. Following the foregoing findings on the importance of six features, we established a simplified and portable decision model defined as “simple-tree XGBoost”. Since 197 subjects had missing detection for at least one of the six critical features, the remaining 787 cases were randomly split into training and internal validation sets in the ratio 7:3 as prior reported [10,11]. Then, simple-tree XGBoost model was re-trained with the same hyperparameters as described above, except for the min_child_weight set to 1 [11]. The performance of the simplified XGBoost model was evaluated in above training and internal validation sets, and also in COVID-19 patients admitted from the Optical Valley Branch of Tongji Hospital (recorded as the external validation set) by assessing the identification accuracy, the precision, recall and F1 scores as described [11]. All participants in the external validation set were included in the XGBoost model since it handled missing values optimally by applying the sparsity-aware algorithm [8]. The AUC scores calculated by simple-tree (six features) or multi-tree (all features) XGboost models and multivariable logistic regression models with all or six features were also separately evaluated in the three datasets.

## Results

### Characteristics of patients from two branches in Wuhan Tongji hospital

As shown in Table 1, the COVID-19 patients from the Sino French New City Branch of Wuhan Tongji Hospital had a median age of 63.0 years old (IQR, 51.0–70.3) and the median days from onset of illness to hospitalization was 11 days (IQR, 7–18), among whom 488 (49.59%) were males. The 286 COVID-19 patients from Optical Valley Branch of Wuhan Tongji Hospital had median age of 60.0 years old (IQR, 44.0–68.0) and the median days from onset of illness to hospitalization of 15 days (IQR, 8–28), and 146 (51.05%) of them were males. Among both two populations, fever was the most frequent onset symptoms, followed by cough (65.14%) (Table 1). The top 3 comorbidities were hypertension, diabetes, cardio-cerebral-vascular disease (CCVD) for patients admitted in the both Branches. Compared to the survivors, the non-survivors were elder, more males, and had a higher proportion of severe symptoms (all *p* < .05) (Table 1). Laboratory testing results for patients from the two branches are showed in Table 2, while the detectable rate and variable types of laboratory findings used in our analysis are presented in Supplementary Table 1.

**Table 1.** Demographic and clinical characteristics among COVID-19 patients admitted in two branches of Wuhan Tongji hospital.

Characteristics	COVID-19 patients from the Sino French New City Branch				COVID-19 patients from the Optical Valley Branch			
	All	Survivors	Non-survivors	*P* ^a^	All	Survivors	Non-survivors	*P* ^a^
No.	984	919	65		286	279	7	
*Age, years-old*	63 (51-70.25)	62 (50-70)	71 (64-78)	<.001	60 (44-68)	59 (43-67)	83 (68.5-83.5)	.003
*Males, no. /total no.(%)*	488/984 (49.59)	443/919 (48.21)	45/65 (69.23)	.001	146/286 (51.05)	139/279 (49.82)	7/7 (100.00)	.015
*Days from illness onset to hospital admission*	11 (7-18)	11 (7-19)	10 (6-14)	.054	15 (8-28)	16 (8-29)	10 (7.5-17)	.315
*Days from illness onset to outcome*	–	39 (32-50)	27 (18-35)	–	–	50 (39-58)	38 (31.5-48.0)	–
*Days from hospital admission to outcome*	–	25 (17-35.75)	13 (8-13)	–	–	28 (16.0-40.5)	29 (18-35)	–
*Smoking status*								
Ever smoking	26/620 (4.19)	23/570(4.04)	3/50 (6.00)	.458	15/249 (6.02)	15/243 (6.17)	0	–
*Symptoms, no. /total no.(%)*								
No. of symptoms				.789				.236
0	8/984 (0.81)	8/919 (0.87)	0		4/286 (1.40)	4/279 (1.43)	0	
1	208/984 (21.14)	196/919 (21.33)	12/65 (18.46)		73/286 (25.52)	70/279 (25.09)	3/7 (42.86)	
2	375/984 (38.11)	352/919 (38.30)	23/65 (35.39)		140/286 (48.95)	139/279 (49.82)	1/7 (14.29)	
3	264 (26.83)	244/919 (26.55)	20/65 (30.77)		59/286 (20.63)	56/279 (20.07)	3/7 (42.86)	
4	87/984 (8.84)	81/919 (8.81)	6/65 (9.23)		10/286 (3.50)	10/279 (3.58)	0	
5	26/984 (2.64)	23/919 (2.50)	3/65 (4.62)		–	–	–	
6	8/984 (0.81)	7/919 (0.76)	1/65 (1.54)		–	–	–	
7	8/984 (0.81)	8/919 (0.87)	0		–	–	–	
Headache/Dizzy	46/984 (4.68)	43/919 (4.68)	3/65 (4.62)	–	2/286 (0.70)	0	2/7 (28.57)	<.001
Fever	768/984 (78.08)	711/919 (77.37)	57/65 (87.69)	.074	204/286 (71.33)	200/279 (71.69)	4/7 (57.14)	.413
Cough	641/984 (65.14)	600/919 (65.29)	41/65 (63.08)	.82	180/286 (62.94)	177/279 (63.44)	3/7 (42.86)	.43
Dyspnea	179/984 (18.19)	165/919 (17.95)	14/65 (21.54)	.577	39/286 (13.64)	39/279 (13.98)	0	.599
Fatigue	159/984 (16.16)	149/919 (16.21)	10/65 (15.39)	–	38/286 (13.29)	35/279 (12.55)	3/7 (42.86)	.052
Myalgia	64/984 (6.50)	62/919 (6.75)	2/65 (3.08)	.429	8/286 (2.80)	8/279 (2.87)	0	–
Sore throat	26/984 (2.64)	26/919 (2.83)	0	.409	5/286 (1.75)	4/279 (1.43)	1/7 (14.29)	.117
Chest distress/Short breath	226/984 (22.97)	212/919 (23.07)	14/65 (21.54)	.896	40/286 (13.99)	40/279 (14.34)	0	.599
Nausea/Vomiting	28/984 (2.85)	25/919 (2.72)	3/65 (4.62)	.424	7/286 (2.45)	7/279 (2.51)	0	–
Diarrhea/Stomachache	185/984 (18.80)	166/919 (18.06)	19/65 (29.23)	.039	41/286 (14.34)	40/279 (14.34)	1/7 (14.29)	–
*Disease severity*								
Severe	297/984 (30.18)	246/919 (26.77)	51/65 (78.46)	<.001	33/286 (11.54)	29/279 (10.39)	4/7 (57.14)	.004
*Comorbidities*								
Hypertension	345/984 (35.06)	330/919 (35.91)	15/65 (23.08)	.05	98/286 (34.27)	96/279 (34.41)	2/7 (28.57)	–
Diabetes	174/984 (17.68)	167/919 (18.17)	7/65 (10.77)	.179	48/286 (16.78)	47/279 (16.85)	1/7 (14.29)	–
Cardio-cerebral-vascular disease	112/984 (11.38)	105/919 (11.43)	7/65 (10.77)	–	39/286 (13.64)	39/279 (13.98)	0	.599
Malignancies	44/984 (4.47)	41/919 (4.46)	3/65 (4.62)	–	12/286 (4.20)	12/279 (4.30)	0	–
Pulmonary diseases	55/984 (5.59)	52/919 (5.66)	3/65 (4.62)	–	26/286 (9.09)	25/279 (8.96)	1/7 (14.29)	.491
Chronic kidney diseases	32/984 (3.25)	29/919 (3.16)	3/65 (4.62)	.464	6/286 (2.10)	6/279 (2.15)	0	–
Digestive system diseases	54/984 (5.49)	52/919 (5.66)	2/65 (3.08)	.573	22/286 (7.69)	22/279 (7.89)	0	–
Other diseases	41/984 (4.17)	37/919 (4.03)	4/65 (6.15)	.341	20/286 (6.99)	18/279 (6.45)	2/7 (28.57)	.079
*No. of comorbidities* ^b^				.496				.699
0	435/984 (44.21)	402/919 (43.74)	33/65 (50.77)		114/286 (39.86)	111/279 (39.79)	3/7 (42.86)	
1	346/984 (35.16)	321/919 (32.93)	25/65 (38.46)		110/286 (38.46)	106/279 (37.99)	4/7 (57.14)	
2	150/984 (15.24)	144 (15.67)	6/65 (9.23)		45/286 (15.73)	45/279 (16.13)	0	
3	43/984 (4.37)	42/919 (4.57)	1/65 (1.54)		17/286 (5.94)	17/279 (6.09)	0	
4	9/984 (0.92)	9/919 (0.98)	0		0	0	0	
5	1/984 (0.10)	1/919 (0.11)	0		0	0	0	

**Note:** values were shown as median (25^th^, 75^th^ percentiles) for the continuous variables and no. /total no.(%) for the categorical variables.

^a^*p* Values were estimated by using Person Chi-square test or Fisher's exact test for categorical variables and Mann-Whitney *U* test for continuous variables.

^b^The calculation of comorbidity numbers was conducted in seven common diseases including hypertension, diabetes, cardio-cerebral-vascular disease, malignancy, pulmonary disease, chronic kidney disease, and digestive system disease.

**Table 2.** Laboratory findings among COVID-19 patients admitted in two branches of Wuhan Tongji hospital.

Clinical characteristics	COVID-19 patients from the Sino French New City Branch				COVID-19 patients from the Optical Valley Branch			
	All (*n* = 984)	Survivors (*n* = 919)	Non-survivors (*n* = 65)	*p* Value^a^	All (*n* = 286)	Survivors (*n* = 279)	Non-survivors (*n* = 7)	*p* Value^a^
Blood routine examination								
WBC count, ×10^9^/L	5.8 (4.54-7.79)	5.75 (4.5-7.7)	7.27 (5.04-10.42)	.001	5.92 (4.86-7.44)	5.91 (4.86-7.39)	7.9 (7.16-11.07)	.040
Lymphocyte cell count,×10^9^/L	1.04 (0.7-1.48)	1.08 (0.72-1.51)	0.64 (0.45-0.9)	<.001	1.38 (0.98-1.81)	1.41 (1.02-1.82)	0.56 (0.54-0.68)	<.001
Neutrophil cell count, ×10^9^/L	4 (2.83-5.88)	3.91 (2.79-5.65)	6.2 (4.16-8.73)	<.001	3.73 (2.78-5.14)	3.68 (2.77-5.02)	6.41 (5.54-9.89)	.008
Neutrophil-Lymphocyte ratio	3.72 (2.24-7.12)	3.56 (2.13-6.44)	9.19 (5.16-13.5)	<.001	2.58 (1.8-4.33)	2.52 (1.79-3.92)	12.33 (6.74-17.79)	<.001
RBC count, ×10^9^/L	4.13 (3.73-4.57)	4.11 (3.73-4.54)	4.28 (3.82-4.69)	.061	4.19 (3.78-4.59)	4.17 (3.78-4.59)	4.23 (4.1-4.62)	.606
Platelet count, ×10^9^/L	212 (159-275.25)	213 (162-280)	164 (113-248)	<.001	219 (180.25-293)	220 (182.5-293.5)	176 (157.5-203)	.121
Hemoglobin, g/L	127 (115-138)	127 (115-138)	135 (120-147)	.019	128 (118-139)	128 (118-139)	138 (131-144.5)	.140
Cytokines and infection related factors								
IL-10, pg/mL	2.5 (2.5-6.6)	2.5 (2.5-6.2)	10.3 (5.38-17.17)	<.001	2.5 (2.5-2.5)	2.5 (2.5-2.5)	2.5 (2.5-5.55)	.411
≥9.1 pg/mL, no. /total no.(%)	147/869 (16.92)	113/811 (13.93)	34/58 (58.62)	<.001	17/284 (5.99)	16/277 (5.78)	1/7 (14.29)	.354
IL-1beta, pg/mL	2.5 (2.5-2.5)	2.5 (2.5-2.5)	2.5 (2.5-2.5)	.407	2.5 (2.5-2.5)	2.5 (2.5-2.5)	2.5 (2.5-2.5)	.678
≥5 pg/mL, no. /total no.(%)	121/869 (13.92)	115/811 (14.18)	6/58 (10.35)	.536	55/284 (19.37)	54/277 (19.50)	1/7 (14.29)	–
IL-2R, pg/mL	595 (379-925)	577 (370.5-873.5)	1205.5 (777.5-1805.75)	<.001	423 (285.75-732)	417 (285-725)	1031 (758-1071)	.007
IL-6, pg/mL	8.34 (2.6-27.13)	6.94 (2.35-23.84)	57.4 (22.61-128)	<.001	3.68 (1.73-10.28)	3.65 (1.69-9.28)	46.98 (29.16-122.5)	<.001
≥7 pg/mL, no. /total no.(%)	467/876 (53.31)	408/817 (49.94)	59/59 (100.00)	<.001	94/284 (33.10)	87/277 (31.41)	7/7 (100.00)	<.001
IL-8, pg/mL	11.4 (6.5-21.5)	10.6 (6.4-20.1)	26.35 (18.7-58.4)	<.001	10.1 (6.77-14.9)	9.9 (6.6-14.9)	22.2 (11.7-32.7)	.027
≥62 pg/mL, no. /total no.(%)	54/869 (6.21)	40/811 (4.93)	14/58 (24.14)	<.001	10/284 (3.52)	10/277 (2.61)	0	–
TNF-α, pg/mL	7.9 (5.9-10.6)	7.8 (5.8-10.4)	11.4 (7.48-15.78)	<.001	8.25 (6.27-10.53)	8.2 (6.2-10.3)	11 (8.8-11.6)	.118
≥8.1 pg/mL, no. /total no.(%)	418/869 (48.10)	379/811 (46.73)	39/58 (67.24)	.004	152/284 (53.52)	147/277 (53.07)	5/7 (71.43)	.456
ESR, mm/h	34 (18-62)	33 (18-62)	38.5 (27.75-65.5)	.041	15 (7-42)	15 (7-40)	87 (48.5-98.5)	.143
hs-CRP, mg/L	26.95 (4.9-76.97)	24 (4.1-67.7)	100.2 (65-166.3)	<.001	3.9 (1.02-27.35)	3.4 (0.95-26.35)	67.2 (31.55-163.4)	.002
PCT, ng/mL	0.05 (0.03-0.11)	0.05 (0.03-0.1)	0.18 (0.09-0.45)	<.001	0.06 (0.05-0.09)	0.06 (0.05-0.08)	0.33 (0.16-0.62)	<.001
>0.05 ng/mL, no. /total no.(%)	467/945 (49.42)	410/881 (46.54)	57/64 (89.06)	<.001	155/246 (63.01)	148/239 (61.93)	7/7 (100.00)	.049
Blood coagulation factor								
PT, s	14 (13.4-14.6)	14 (13.4-14.5)	14.8 (14.2-15.6)	<.001	13.5 (13-14.1)	13.5 (13-14)	15 (14.15-16)	.005
APTT, s	39.15 (36-43.48)	39.1 (36-43.3)	40.3 (36-46.2)	.037	38.35 (35.6-41.2)	38.3 (35.6-41.05)	41.5 (37.2-48.4)	.203
D-dimer, mg/L	0.93 (0.42-2.04)	0.86 (0.4-1.94)	2.05 (0.96-5.7)	<.001	0.42 (0.22-0.96)	0.42 (0.22-0.93)	5.51 (0.64-21)	.005
INR	1.07 (1.01-1.13)	1.07 (1.01-1.12)	1.15 (1.08-1.22)	<.001	1.04 (0.99-1.1)	1.04 (0.99-1.09)	1.19 (1.1-1.29)	.005
FIB, mg/L	4.77 (3.68-5.88)	4.73 (3.67-5.8)	5.82 (4.16-6.92)	<.001	3.96 (3.12-5.13)	3.89 (3.12-5.03)	5.21 (5.02-5.58)	.046
Serum biochemical index								
hs-cTnI, ng/L	4.65 (2.2-12.25)	4.4 (2.1-10.67)	22.25 (7.03-61.67)	<.001	2.4 (0.95-6.2)	2.2 (0.95-5.65)	25.3 (17.65-173.9)	<.001
M: >34.2, ng/L, no. /total no.(%)	51/423 (12.06)	33/379 (8.71)	18/44 (40.91)	<.001	8/142 (5.63)	5/135 (3.70)	3/7 (42.86)	.004
F: >15.6, ng/L, no. /total no.(%)	75/439 (17.08)	63/419 (15.04)	12/20 (60.00)	<.001	15/136 (11.03)	15/136 (11.03)	–	–
ALT, U/L	24 (15-40)	24 (15-40)	27 (15-38)	.774	23 (15-39.75)	23 (14.5-39)	39 (22-43)	.257
AST, U/L	27 (20-40)	26.5 (20-39)	41 (26-61)	<.001	23 (18-33)	22 (18-32)	38 (32.5-40.5)	.004
Albumin, g/L	34.8 (31.3-38.8)	35.1 (31.6-39.08)	30.8 (28.3-34)	<.001	38.7 (33.4-42.68)	38.9 (33.7-42.7)	33.3 (31.55-35.5)	.091
TBIL, μmol/L	9.3 (6.8-12.65)	9.15 (6.7-12.47)	11.6 (8.8-15.1)	<.001	8.4 (6.1-11.95)	8.2 (6.05-11.7)	12.3 (11.8-25.25)	.003
Cr, μmol/L	69 (57-84)	68 (56-83)	85 (64-104)	<.001	68.5 (57-83.75)	68 (57-82.5)	107 (89.5-138)	.001
BUN, mmol/L	4.6 (3.5-6.1)	4.5 (3.4-5.8)	7.4 (5-10)	<.001	4.5 (3.7-5.6)	4.5 (3.65-5.5)	11.9 (9.4-13.3)	<.001
LDH, U/L	274 (217-368.5)	268 (215-351)	443 (324-599)	<.001	210.5 (170-301.25)	207 (169.5-295)	408 (347.5-460.5)	.001
eGGR^b^, ml/min/1.73m^2^	91.6 (77.25-102.5)	92.25 (78.82-103.05)	79 (50.8-92.9)	<.001	94.35 (78.42-105.1)	94.8 (80.2-105.4)	55 (46.45-68.4)	.001
eGFR (MDRD)^c^, ml/min/1.73m^2^	102.92 (83.42-124.53)	104 (85.76-125.18)	81.25 (59.61-104.69)	<.001	106.78 (83.73-124.35)	107.17 (85.07-125.05)	62.69 (47.98-79.69)	.001
GLU, mmol/L	6.24 (5.33-7.81)	6.16 (5.3-7.7)	7.3 (6.18-9.41)	<.001	5.54 (4.93-6.94)	5.53 (4.89-6.71)	8.89 (7.05-11.15)	.001
NT-proBNP, pg/mL	143 (52-466)	122 (46.5-414)	505.5 (232-1008.5)	<.001	68 (22-224.25)	62 (21-191)	1099 (328.5-1360)	.001
Serum ferritin, μg/L	576.4 (348.2-1101.42)	548.95 (325.28-947.7)	1427.55 (857.18-2398.22)	<.001	342.8 (173.3-673.3)	314 (166.5-615.9)	1851.45 (1198.2-2351.55)	.001

WBC: white blood cell count; RBC: red blood cell count; hs-CRP: high-sensitivity C-reactive protein; PCT: procalcitonin; ESR: erythrocyte sedimentation rate; IL-10: interleukin-10; IL-1β: Interleukin-1beta; IL-2R: Interleukin-2 receptor; IL-6: Interleukin-6; IL-8: Interleukin-8; TNF-α: tumour necrosis factor-α; PT: prothrombin time; APTT: activated partial thromboplastin time; FIB: fibrinogen; INR: international normalised ratio; hs-cTnI: hypersensitive cardiac troponin I; NT-proBNP: N-terminal pronatriuretic peptide; GLU: glucose; LDH: lactate dehydrogenase; ALT: alanine aminotransferase; AST: aspartate aminotransferase; TBIL: total bilirubin; Cr: creatinine; BUN: blood urea nitrogen; eGFR: glomerular filtration rate.

Continuous variables were shown as median (25th, 75th percentiles).

^a^*p* Values were estimated by using Person Chi-square test or Fisher's exact test for categorical variables and Mann-Whitney *U* test for continuous variables. ^b^eGFR was calculated by CKD-EPI equation.

^c^eGFR was calculated by abbreviated MDRD equation.

### Clinical features selection in LASSO regression analysis

A total of 48 clinical features detected at hospital admission were enter into the LASSO regression analysis, and 19 were significantly associated with COVID-19 death risk, including age, gender, disease severity, number of symptoms, comorbidities of CCVD, hypertension, diabetes, chronic kidney disease, number of comorbidities, blood count of neutrophils, level of activated partial thromboplastin time (APTT), high-sensitivity C-reactive protein (hs-CRP), lactate dehydrogenase (LDH), serum ferritin, and abnormal level of hypersensitive troponin I (hs-cTnI), interleukin-6 (IL-6), IL-8, IL-10, and IL-1β (Supplementary Figure 1).

### Features importance for an operable decision model

The aforementioned 19 features were entered into multi-tree XGBoost and top 10 clinical features were ranked by this model based on the values of their importance (Supplementary Figure 2). Subsequently, we added the ranked features one by one to the XGBoost model until an AUC score improving inferior to 0.5%. Six features, including disease severity, hs-CRP, age, LDH, serum ferritin, IL-10, were selected as the significant factors (Table 3). Application of the multi-tree XGBoost algorithm with aforesaid six features resulted in a mean AUC (SD) of 0.921 (0.038) and 0.891 (0.053) among training and internal validation sets respectively, suggesting that this model was accurate enough to discriminate the deceased outcome of patients (Table 3).

**Table 3.** Performance of the multi-tree XGBoost classification in discriminating death outcomes by using 100-round fivefold cross-validation among COVID-19 patients admitted in the Sino French New City Branch of Wuhan Tongji Hospital.

Variables	AUC scores (mean ± SD)	
	Training set	Internal validation set
Severity	0.779 ± 0.037	0.660 ± 0.083
Severity, hs-CRP	0.863 ± 0.046	0.732 ± 0.120
Severity, hs-CRP, Age	0.887 ± 0.035	0.785 ± 0.082
Severity, hs-CRP, Age, LDH	0.900 ± 0.039	0.825 ± 0.094
Severity, hs-CRP, Age, LDH, Serum ferritin	0.911 ± 0.051	0.868 ± 0.062
Severity, hs-CRP, Age, LDH, Serum ferritin, IL-10	0.921 ± 0.038	0.891 ± 0.053
Severity, hs-CRP, Age, LDH, Serum ferritin, IL-10, APTT	0.907 ± 0.039	0.867 ± 0.079

### Construction and evaluation of simple-tree XGBoost model

**Figure 1.**
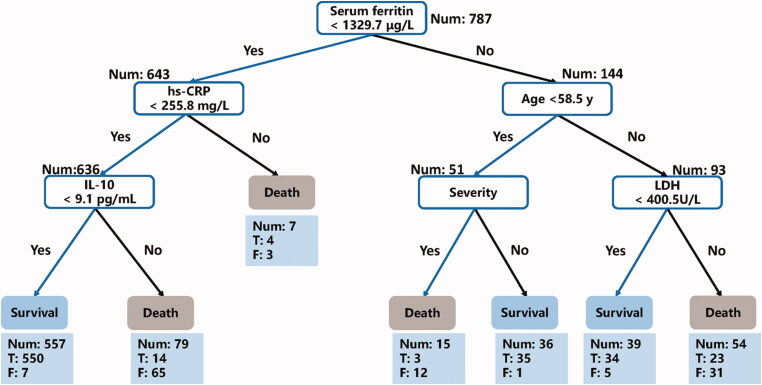
A decision rule using 6 key clinical features and their thresholds in absolute values among COVID-19 patients admitted in the Sino French New City Branch of Wuhan Tongji Hospital (*n* = 787). Num: the number of patients in a class; T: the number of correctly classified; F: the number of misclassified patients.

Then a simple-tree XGBoost model was constructed based on the above six key features. The performance of the simple-tree XGBoost among COVID-19 patients were presented in Table 4. In the training set, we observed a 99.2% survival and a 100% death prediction precision, and the recalls of survival and death prediction were 100% and 90.2%, respectively; in the internal validation set, the precisions of survival and death prediction showed 99.1% and 100%, respectively, while the survival and death prediction recalls were 100% and 87.5% separately. Similar results were observed in the external validation set, manifesting 99.6% and 100% prediction precision of survival and decease separately, as well as 100% survival prediction recall and 85.7% death prediction recall. In general, the F1 scores including survival and decease prediction, accuracy, weighted and macro averages are all >0.90 among COVID-19 patients in the three sets (Table 4). Moreover, one of the decision trees structure illustrated with the aforementioned six features was presented in Figure 1.

**Table 4.** Performance of the proposed simple-XGBoost algorithm by using 6 key clinical features among COVID-19 patients.

Datasets	No. of patients	Precision	Recall	F1-score
Training set (554 cases from the Sino French New City Branch of Tongji Hospital)				
Survival	513	0.992	1	0.996
Death	41	1	0.902	0.949
Accuracy	554	–	–	0.993
Macro averages	554	0.996	0.951	0.973
Weighted averages	554	0.993	0.993	0.993
Internal validation set (233 cases from the Sino French New City Branch of Tongji Hospital)				
Survival	217	0.991	1	0.995
Death	16	1	0.875	0.933
Accuracy	233	–	–	0.991
Macro averages	233	0.995	0.938	0.964
Weighted averages	233	0.991	0.991	0.991
External validation set (286 cases from the Optical Valley Branch of Tongji Hospital)				
Survival	279	0.996	1	0.998
Death	7	1	0.857	0.923
Accuracy	286	–	–	0.997
Macro averages	286	0.998	0.929	0.961
Weighted averages	286	0.996	0.997	0.996

**Figure 2.**
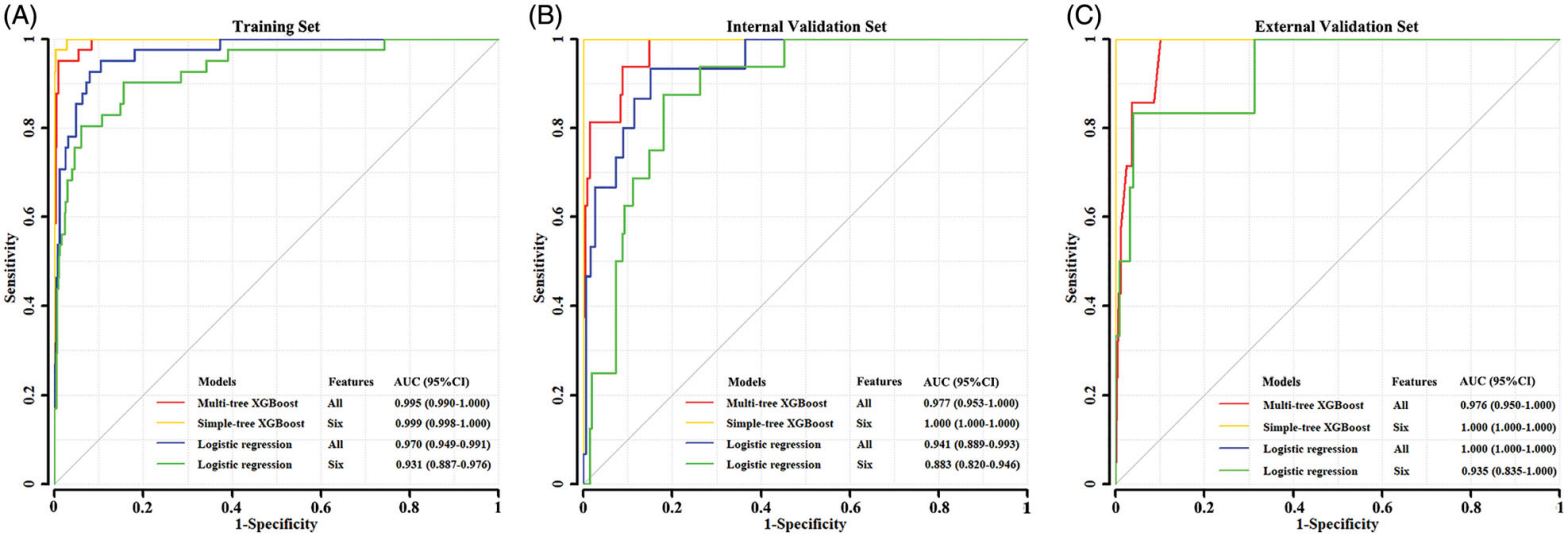
ROC curves for COVID-19 patients in the training (A) and internal validation (B) sets from Sino French New City Branch of Wuhan Tongji hospital (*n* = 787) and in the external validation set (C) from Optical Valley Branch of Wuhan Tongji Hospital (*n* = 286). Since 197 subjects with missing detection for at least one of the 6 features, the remaining 787 cases were randomly split into training (*n* = 554) and internal validation sets (*n* = 233) in the ratio 7:3.

For the benchmark purpose, we also compared the performances of XGBoot model with the conventional multivariable logistic regression model. In the training set, the simple-tree XGBoost model with 6 selected features revealed superior performance compared to the logistic regression with all 19 features (AUC: 0.999 vs. 0.970, *p* = .008) or 6 features (AUC: 0.999 vs. 0.931, *p* = .003) (Figure 2(A)), while no significant difference in AUC score was observed between simple-tree and multi-tree models (AUC: 0.999 vs 0.995, *p* = .056) (Figure 2(A)). Similarly, in internal validation set, the simple-tree XGBoost model exhibited better performance than the logistic regression used by all 19 features (AUC: 1.000 vs. 0.941, *p* = .026) or the six selected features (AUC: 1.000 vs. 0.883, *p* < .001), as well as showing marginal higher AUC compared to multi-tree XGBoost model (AUC: 1.000 vs 0.977, *p* = .049) (Figure 2(B)). In the external validation set, the simple-tree XGBoost model by using six selected features and logistic regression model by using 19 features showed a superior performance (both AUC = 1.000, Figure 2(C)). Briefly, the above results suggested that simple-tree XGBoost model owned more precise and stable prediction performance than multivariable logistic regression in identifying fatal outcome of patients.

## Discussion

The retrospective cohort study conducted in the COVID-19 patients hospitalized in two branches of Wuhan Tongji hospital demonstrated that six features including disease severity, age, and serum levels of hs-CRP, LDH, ferritin, and IL-10 were significant predictors for death risk of COVID-19 patients. The defined simple-tree XGBoost model constructed with these six features revealed satisfactory performance with the AUC scores higher than 99% in training and internal validation set, and prediction precisions of survival and death were both >95% in the external validation set.

Judging from the feature importance ranked by XGBoost model, the disease severity is the most crucial factor for death risk prediction. Recently, several studies have reported 41.1–61.5% hospital fatality rate of critical patients, which is significantly higher than fatality rate of 1.1–1.7% among mild and moderate patients [2,12–14]. Patients with severe manifestations should be paid much attention to get appropriate treatment approaches and further reducing their death risk. Our study also emphasised that the elder patients had a higher death risk, which was consistent with many previous studies [15,16].

Using laboratory biomarkers to construct prediction models is a comprehensive and efficient method for identifying progression towards severity and fatal outcomes of COVID-19. In our study, four biomarkers, namely hs-CRP, LDH, serum ferritin, and IL-10, were selected as risk factors for death prediction. The hs-CRP, a crucial biomarker described in prior COVID-19 studies for undesirable prognosis in ARDS, revealed an enduring status of inflammation [17,18], which might deeply interact with inflammatory storm, causing lung damage and pulmonary oedema of patients with COVID-19 [19,20]. LDH is regarded as an important and ubiquitous cellular enzyme, and the serum level of LDH has been identified as a significant biomarker for lung fibrosis and infection [21,22]. Previous studies have also reported the relationship between increasing LDH and higher death risk of COVID-19 [4,11,23]. Ji *et al.* also observed that COVID-19 patients with serum level of LDH higher than 500 U/L showed a significant illness progression hazard ratio of 9.8 (*p* < .001) in a multivariate Cox analysis, when compared to the group with LDH level <250 U/L [24].

dOne prior meta-analysis has recommended serum ferritin and IL-10 as candidate biomarkers for predicting COVID-19 progression to critical illness [25]. But few epidemiological studies provided direct support for the associations of serum ferritin and IL-10 with COVID-19 fatal risk. An early case–control study reported that appraising serum levels of ferritin in subjects at risk for and with ARDS may contribute to predict progression of ARDS and thereby improve relevant treatment approach [26]. Wu *et al.* conducted a retrospective cohort study among 201 COVID-19 patients and found that elevated serum ferritin was an independent risk factor related to ARDS development, but similar association was not observed when examined for death outcome, possibly due to a limited sample size [27]. Another case–control study conducted among 144 COVID-19 patients in Italy reported that patients who died during the hospital stay presented significant higher level of serum ferritin, but the multiple regression analysis by incorporating clinical and laboratory variables abolished the significant association of serum ferritin with in-hospital death [28]. Interestingly, one study (*n* = 174) focussed on ferritin level with regard to existing comorbidities, such as diabetes, found that diabetes patients with confirmed COVID-19 had a higher median level of ferritin than non-diabetics patients of COVID-19 (764.8 vs. 128.9 µg/L, *p* < .001), revealing that diabetics suffering from COVID-19 may face a higher probability to generate inflammation and might experience serious complications from COVID-19 [29]. An *in vitro* study conducted in human hepatoblastoma cell line HepG2 reported that the inflammation-related cytokines (e.g. IL-1β and IL-6) might elevate ferritin synthesis [30]. Therefore, cytokines induced by COVID-19, which are generally increased in infected patients, might unite serum ferritin production in early inflammation and further result in patient’s worse prognosis, but the underlying mechanisms need to be further elucidated. IL-10 is now described as a complex anti-inflammation cytokine generated by different cell types, showing vital effect in regulating immune and inflammation responses [31]. Han *et al.* performed a case-control study by using a series of inflammation markers (e.g. IL-6, IL-10, and TNF-α) among 102 COVID-19 patients and finally highlighted the significance of IL-6 and IL-10 as illness severity predictors [32]. Another longitudinal analysis conducted in 71 COVID-19 patients revealed that a combination of IL-10, RANTES, and IL-1 receptor antagonist at first week of follow-up might be useful prediction biomarkers for patients’ outcome [33]. Increasing of IL-10 in severe patients might be related to a compensatory anti-inflammatory response, which may lead to higher proportion of subsequent infections, sepsis, and further raising the death risk [34]. Although possible biological relationships exist between the above biomarkers (hs-CRP, LDH, serum ferritin, and IL-10) and severity of COVID-19, the effects of these biomarkers in COVID-19 pathogenesis still need validations and further in-depth investigations.

Though the common use of ML method in business analysis, the mainstream medical domain has still fell behind in terms of studying and applying ML method for real-time risk prediction. In this study, the XGBoost method exhibited superior and stable performance in COVID-19 mortality risk prediction. Prior comparison studies have revealed that ML methods can be more accurate and efficient than traditional logistic regression analysis, especially when the sample size was limited [35]. A prospective study conducted in 38 women breast cancer patients used several ML methods with multiparametric magnetic resonance imaging, to make early prediction of pathological complete response (pCR) to neoadjuvant chemotherapy and of survival outcomes, observed that, of all ML classifier model, the XGBoost model outperformed all other models such as linear support vector machine, logistic regression, and random forests in the prediction of pCR (with mean AUC of 0.8577 and best AUC of 0.9430) [36].

Our study provided a portable and intuitive clinical proof to accurately identify the death risk of patients with COVID-19 by using an efficient ML method. A prior study conducted in Wuhan COVID-19 patients also used the XGBoost model to explore the death risk factors, but the 375 patients they included in the training set had a higher proportion of critical clinical symptoms (40.3%) [11], probably because they mainly included the COVID-19 patients admitted in the Department of Critical Care Medicine and this rate was much higher than the Wuhan report (critical rate 3.0%) [37]. They reported a mortality rate of 46.4% and only identified three features (LDH, lymphocyte and hs-CRP) to be significant death risk factors, while our present study in a larger number of COVID-19 patients with a case-fatality rate of 5.67% (close to the mortality rate to date in Wuhan: 7.68%) [38], could give a better representative of the general patients. Additionally, by using both LASSO model and XGBoost ML algorithm, we were able to validate previous results like age, disease severity, hs-CRP, and LDH were significant death risk markers for COVID-19 and further provide direct evidence for the fatal effects of serum ferritin and IL-10. However, several limitations should also be noted. Firstly, we constructed the XGBoost model with a modest sample size, while sample size for external validation was comparatively small. Nevertheless, the performance of death risk prediction performed in XGBoost model was superior in the two populations. Secondly, the proposed ML method is absolutely data-driven, which might be influenced by class imbalance resulting from low fatal rate and further disturbed prediction accuracy and sensitivity. Hence, larger scale and multicentre validation studies with improved data balance should be completed to obtain stable prediction effect and extent to varied dataset rationally. Thirdly, the dataset for model construction and validation are entirely from China, which might restrict the generalizability of the ML model to the other areas of the world. Finally, the classification capacity of ML method still needs to be improved by balancing the association between model interpretability and prediction accuracy. Clinicians obviously show preference to comprehensible method like logistic model, but a black-box model might present preferable performance.

## Conclusions

In this study, we identified six candidate features, including disease severity, age, and serum levels of hs-CRP, LDH, ferritin, and IL-10 measured at hospital admission, as critical death risk biomarkers for COVID-19 patients. The simple-tree XGBoost ML model conducted by the six significant features can help to predict death risk of hospitalized COVID-19 patients accurately with >90% precision and >85% sensitivity. Since the six key features were generally detectable at hospital admission, early monitoring of these features might help to prioritise high risk COVID-19 patients and optimise the limited medical resources during the pandemic period.

## Supplementary Material

Supplemental Material

## Data Availability

Data sharing is not applicable to this paper as the datasets generated needed to be confidential.
