# Supplementary material for: Clinical and inflammatory features based machine learning model for fatal risk prediction of hospitalized COVID-19 patients: results from a retrospective cohort study
Source: Ann Med. 2021 Jan 7;53(1):257–66. doi: 10.1080/07853890.2020.1868564 (PMC7799376; doi:10.1080/07853890.2020.1868564)
Supplement: Supplemental Material [file IANN_A_1868564_SM3607.docx]

| **Supplementary Table 1. The detectable rate and variable types of laboratory findings used in this study.** | | | | | | | |
| --- | --- | --- | --- | --- | --- | --- | --- |
| **Variables** | **All (n=1270)** | | Variable types used in the analyses models | **Sino French New City Branch (n=984)** | | **Optical Valley Branch (n=286)** | |
|  | Missing,  no. (%) | < LOD,  no./total no. (%) |  | Missing,  no. (%) | < LOD,  no./total no. (%) | Missing,  no. (%) | < LOD,  no./total no. (%) |
| ***Blood routine examination*** |  |  |  |  |  |  |  |
| WBC count, ×10^9^/L | 0 | 0 | continuous | 0 | 0 | 0 | 0 |
| Lymphocyte cell count,×10^9^/L | 0 | 0 | continuous | 0 | 0 | 0 | 0 |
| Neutrophil cell count, ×10^9^/L | 0 | 0 | continuous | 0 | 0 | 0 | 0 |
| Neutorphil-Lymphocyte ratio | 0 | 0 | continuous | 0 | 0 | 0 | 0 |
| RBC count, ×10^9^/L | 0 | 0 | continuous | 0 | 0 | 0 | 0 |
| Platelet count, ×10^9^/L | 4 (0.31) | 0 | continuous | 4 (0.41) | 0 | 0 | 0 |
| Haemoglobin, g/L | 0 | 0 | continuous | 0 | 0 | 0 | 0 |
| ***Cytokines and infection related factors*** |  |  |  |  |  |  |  |
| IL-10, pg/mL | 117 (9.21) | 789/1153 (68.43) | binary | 115 (11.69) | 561/869 (64.56) | 2 (0.7) | 228/284 (80.28) |
| IL-1beta, pg/mL | 117 (9.21) | 977/1153 (84.74) | binary | 115 (11.69) | 748/869 (86.08) | 2 (0.7) | 229/284 (80.63) |
| IL-2R, pg/mL | 117 (9.21) | 4/1153 (0.35) | continuous | 115 (11.69) | 2/869 (0.23) | 2 (0.7) | 2/284 (0.70) |
| IL-6, pg/mL | 110 (8.66) | 195/1160 (16.81) | binary | 108 (10.98) | 137/876 (15.64) | 2 (0.7) | 58/284 (20.42) |
| IL-8, pg/mL | 117 (9.21) | 183/1153 (15.87) | binary | 115 (11.69) | 146/869 (16.80) | 2 (0.7) | 37/284 (13.03) |
| TNF-α, pg/mL | 117 (9.21) | 109/1153 (9.45) | continuous | 115 (11.69) | 88/869 (10.13) | 2 (0.7) | 21/284 (7.39) |
| ESR, mm/h | 345 (27.17) | 0 | continuous | 159 (16.16) | 0 | 186 (65.03) | 0 |
| hs-CRP, mg/L | 22 (1.73) | 2/1248 (1.60) | continuous | 22 (2.24) | 2/962 (2.08) | 0 (0) | 0 |
| PCT, ng/mL | 79 (6.22) | 177/1191 (14.86) | binary | 39 (3.96) | 176/945 (18.62) | 40 (13.99) | 1/246 (0.41) |
| ***Blood coagulation factor*** |  |  |  |  |  |  |  |
| PT, s | 13 (1.02) | 0 | continuous | 9 (0.91) | 0 | 4 (1.4) | 0 |
| APTT, s | 54 (4.25) | 0 | continuous | 50 (5.08) | 0 | 4 (1.4) | 0 |
| D-dimer, mg/L | 13 (1.02) | 61/1257 (4.85) | continuous | 9 (0.91) | 57/975 (5.85) | 4 (1.4) | 0 |
| INR | 13 (1.02) | 0 | continuous | 9 (0.91) | 0 | 4 (1.4) | 0 |
| FIB, mg/L | 53 (4.17) | 0 | continuous | 49 (4.98) | 0 | 4 (1.4) | 0 |
| ***Serum biochemical index*** |  |  |  |  |  |  |  |
| hs-cTnI, ng/L | 130 (10.24) | 300/1140 (26.32) | binary | 122 (12.4) | 181/862 (20.80) | 8 (2.8) | 119/278 (42.81) |
| ALT, U/L | 1 (0.08) | 1/1269 (0.079) | continuous | 1 (0.1) | 0 | 0 | 1/286 (0.35) |
| AST, U/L | 1 (0.08) | 0 | continuous | 1 (0.1) | 0 | 0 | 0 |
| Albumin, g/L | 5 (0.39) | 0 | continuous | 5 (0.51) | 0 | 0 | 0 |
| TBIL, μmol/L | 5 (0.39) | 1/1265 (0.079) | continuous | 5 (0.51) | 1/979 (0.10) | 0 | 0 |
| Cr, μmol/L | 1 (0.08) | 0 | continuous | 1 (0.1) | 0 | 0 | 0 |
| BUN, mmol/L | 1 (0.08) | 0 | continuous | 1 (0.1) | 0 | 0 | 0 |
| LDH, U/L | 1 (0.08) | 0 | continuous | 1 (0.1) | 0 | 0 | 0 |
| eGGR, ml/min/1.73m^2^ | 11 (0.87) | 0 | continuous | 9 (0.91) | 0 | 2 (0.7) | 0 |
| eGFR (MDRD), ml/min/1.73m^2^ | 1 (0.08) | 0 | continuous | 1 (0.1) | 0 | 0 | 0 |
| GLU, mmol/L | 25 (1.97) | 0 | continuous | 25 (2.54) | 0 | 0 | 0 |
| NT-proBNP, pg/mL | 201 (15.83) | 41/1069 (3.84) | continuous | 179 (18.19) | 32/805 (3.98) | 22 (7.69) | 9/264 (3.41) |
| Serum ferritin, μg/L | 319 (25.12) | 1/ 951 (0.11) | continuous | 164 (16.67) | 1/820 (0.12) | 155 (54.2) | 0 |
| **Abbreviations**: WBC, white blood cell count; RBC, red blood cell count; hs-CRP, high-sensitivity C-reactive protein; PCT, procalcitonin; ESR, erythrocyte sedimentation rate; IL-10, interleukin-10; IL-1β, Interleukin-1beta; IL-2R, Interleukin-2 receptor; IL-6, Interleukin-6; IL-8, Interleukin-8; TNF-α, tumor necrosis factor-α; PT, prothrombin time; APTT, activated partial thromboplastin time; FIB, fibrinogen; INR, international normalized ratio; hs-cTnI, hypersensitive cardiac troponin I;NT-proBNP, N-terminal pronatriuretic peptide; GLU, glucose; LDH, lactate dehydrogenase; ALT, alanine aminotransferase; AST, aspartate aminotransferase; TBIL, total bilirubin; Cr, creatinine; BUN, blood urea nitrogen; eGFR, glomerular filtration rate | | | | | | | |


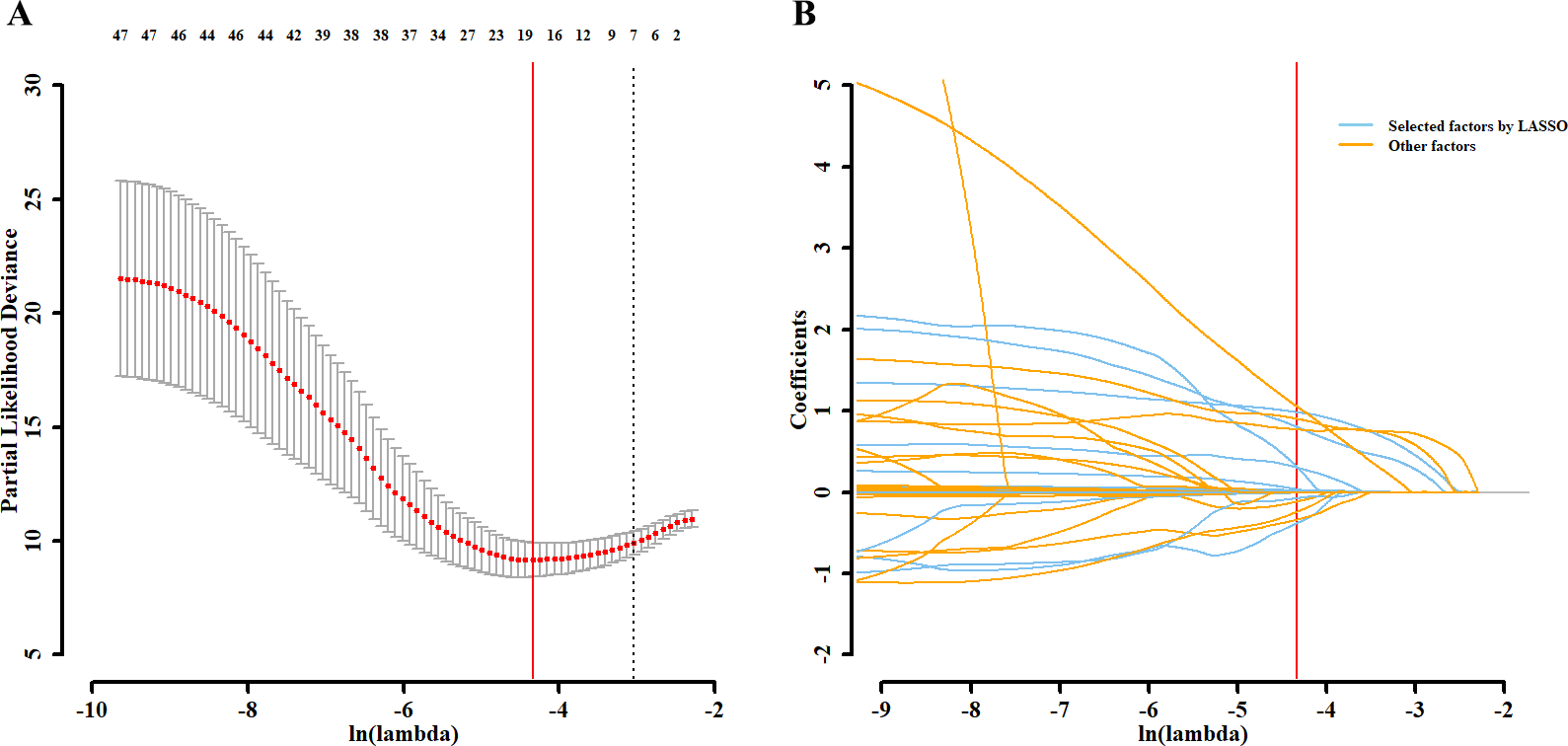


**Supplementary Fig. 1.** Feature selection using the least absolute shrinkage and selection operator (LASSO) Cox regression model.

(A) LASSO coefficient profiles of the 48 baseline clinical features.

(B) Tuning parameter (λ) selection in the LASSO model used 10-fold cross-validation via minimum partial likelihood deviance.


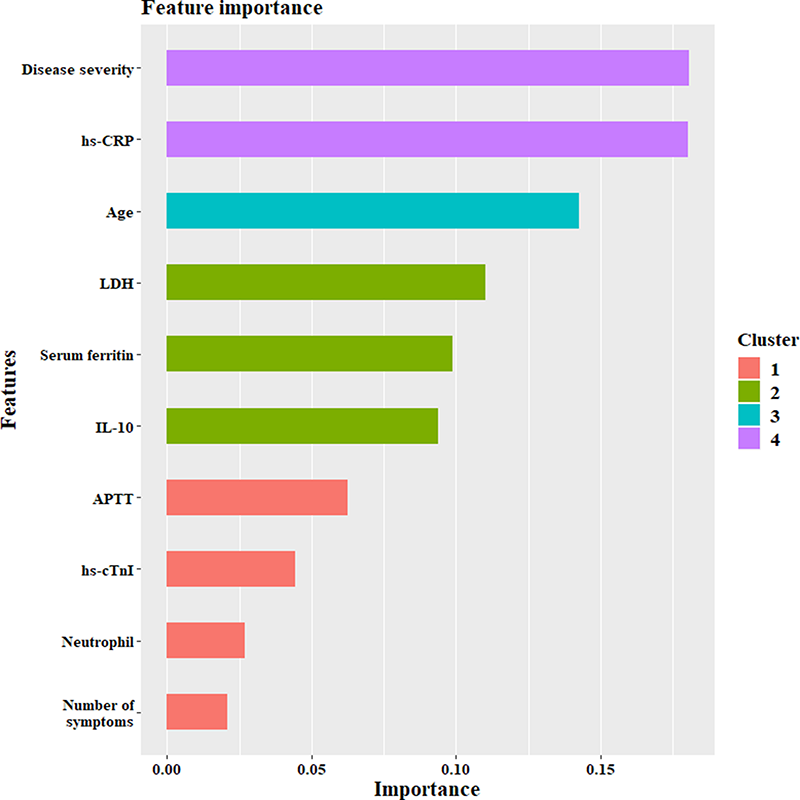


**Supplementary Fig. 2.** The ranks of the clinical features significantly associated with Covid-19 death risk according to their importance in the multi-tree XGBoost algorithm in all 984 subjects admitted in the Sino French New City Branch of Tongji Hospital in Wuhan.

**Abbreviations:** hs-CRP, high-sensitivity C-reactive protein; LDH, lactate dehydrogenase; IL-10, interleukin-10; APTT, activated partial thromboplastin time; hs-cTnI, hypersensitive cardiac troponin I.
